# Supplementary material for: A Principal Component Analysis of 39 Scientific Impact Measures
Source: PLoS One. 2009 Jun 29;4(6):e6022. doi: 10.1371/journal.pone.0006022 (PMC2699100; doi:10.1371/journal.pone.0006022)
Supplement: Appendix S1 — (0.06 MB PDF) [file pone.0006022.s001.pdf]

Appendix S1

Measure definitions

Let $D = (V, E)$ be a directed graph with the set of nodes V and the set of oriented edges E. Assume the edges $e = (u, v)$ have weights $w(u, v) > 0$, $(u, v) \in E$ and $w(u, v)) = 0$ for $(u, v) \notin E$. For unweighted graphs $w(u, v) = 1$, $e(u, v) \in E$.

A undirected graph $G = (V, E)$ can be constructed from D by replacing every oriented edge (u, v) by an undirected edge (u, v) with weight $\hat{w}(u, v) = [w(u, v) + w(v, u)]/2$. For unweighted graphs this amounts to replacing each directed link by an undirected link.

We use the following definitions of network measures:

Degree Centrality

The degree centrality of a node u is defined as the sum of weights of edges incident to u

$$C_D(u) = \sum_{(u,v) \in E} w(u, v)\,. \tag{1}$$

For directed graphs there is an indegree

$$C_{D_{in}}(u) = \sum_{(v,u) \in E} w(v, u)\,, \tag{2}$$

and outdegree

$$C_{D_{out}}(u) = \sum_{(u,v) \in E} w(u, v)\,. \tag{3}$$

Closeness Centrality

Let $d_G(s, t)$ be the shortest distance between nodes s and t in G (the sum of path weight is minimum).

The closeness centrality [1] of a node u is

$$C_C(u) = \frac{1}{\sum_{t \in V} d_G(u, t)}. \tag{4}$$

We use the algorithm from Ref. [2].

Betweenness Centrality

Let σ_{st} be the number of weighted shortest paths between nodes s and t in the graph and $\sigma_{st}(u)$ be the number of those shortest paths that pass through node u. The betweenness centrality [3] is

$$C_B(u) = \sum_{s \neq u \neq t \in V} \frac{\sigma_{st}(u)}{\sigma_{st}}. \tag{5}$$

We use the algorithm from Ref. [2].

PageRank

The PageRank [4] of node v is

$$PR(u) = (1 - d) + d \sum_{(v,u) \in E} w(v, u) \frac{PR(v)}{C_{D_{out}}(v)}\,. \tag{6}$$

See the review [5] for implementation details.

Measure definitions

For a directed graph $D = (V, E)$ with weights $w(u, v)$

Directed weighted

- Directed Weighted In-degree Centrality: Eq. (2)
- Directed Weighted Out-degree Centrality: Eq. (3)
- Directed Weighted Pagerank: Eq. (6)

Directed Unweighted

With $w(u, v) = 1$ for $(u, v) \in E$.

- Directed Unweighted In-degree Centrality: Eq. (2)
- Directed Unweighted Out-degree Centrality: Eq. (3)
- Directed Unweighted Pagerank: Eq. (6)

Undirected weighted

Replacing every oriented edge $(u, v) \in E$ by an undirected edge (u, v) with weight $\hat{w}(u, v) = [w(u, v) + w(v, u)]/2$.

- Undirected Weighted LCC* Betweenness: Eq. (5)
- Undirected Weighted LCC Closeness: Eq. (4)
- Undirected Weighted LCC Out-Degree Centrality: Eq. (1)
- Undirected Weighted LCC Pagerank: Eq. (6)

$\star$: Largest Connected Component

Undirected unweighted

Replacing every oriented edge $(u, v) \in E$ by an undirected edge (u, v) with weight $\hat{w}(u, v) = 1$.

- Undirected Unweighted LCC Betweenness: Eq. (5)
- Undirected Unweighted LCC Closeness: Eq. (4)
- Undirected Unweighted LCC Out-degree Centrality: Eq. (1)
- Undirected Unweighted LCC Pagerank: Eq. (6)

References

1. Sabidussi G (1966) The centrality index of a graph. Psychometrika 31: 581–603.
2. Brandes U (2001) A faster algorithm for betweenness centrality. Journal of Mathematical Sociology 25: 163–177.
3. Freeman LC (1977) Set of measures of centrality based on betweenness. Sociometry 40: 35 – 41.
4. Brin S, Page L, Motwami R, Winograd T (1999) The PageRank citation ranking: bringing order to the Web. Technical Report 1999-0120, Stanford University.
5. Langville AN, Meyer CD (2005) A survey of eigenvector methods for web information retrieval. SIAM Review 47: 135–161.
